# CO_2_ Laser Versus Surgical Deroofing for the Treatment of Hidradenitis Suppurativa Tunnels: A Comparative Multicentric, Retrospective Study

**DOI:** 10.1097/DSS.0000000000004498

**Published:** 2024-11-19

**Authors:** Andrea Sechi, Raffaele Dante Caposiena Caro, Alessandra Michelucci, Valentina Dini, Stefano Piaserico, Iris Zalaudek, Francesco Savoia, Jacopo Tartaglia

**Affiliations:** * Dermatology Unit, San Bortolo Hospital, Vicenza, Italy;; † Division of Dermatology, Department of Experimental, Diagnostic and Specialty Medicine, Azienda Ospedaliero-Universitaria di Bologna Policlinico Sant'Orsola-Malpighi, Bologna, Italy;; ‡ Dermatology Clinic, Hospital Maggiore of Trieste, University of Trieste, Trieste, Italy;; § Department of Dermatology, University of Pisa, Pisa, Italy;; ‖ Interdisciplinary Center of Health Science, Sant'Anna School of Advanced Studies of Pisa, Pisa, Italy;; ¶ Dermatology Unit, Department of Medicine, University of Padova, Padua, Italy;; ** Skin Cancer Unit, IRCCS Istituto Romagnolo per lo Studio dei Tumori (IRST) “Dino Amadori”, Meldola, FC, Italy

## Abstract

**BACKGROUND:**

Tunnels of hidradenitis suppurativa (HS) are one of the most challenging aspects to manage, and different surgical techniques have been proposed for their treatment. CO_2_ laser and surgical deroofing are 2 of the most widely used techniques, but no studies have compared them directly.

**OBJECTIVE:**

The objective of this study was to compare the efficacy and outcomes of CO_2_ laser treatment versus surgical deroofing for HS tunnels, with a focus on healing time, complication rates, pain perception, and cosmetic outcomes.

**MATERIALS AND METHODS:**

The authors performed a multicentric retrospective analysis of 20 patients with HS tunnels who were treated with either CO_2_ laser (*n* = 10) or surgical deroofing (*n* = 10). The primary end point was to compare the 2 procedures in terms of healing time, complication rates, pain, and cosmetic outcome. Outcome measures included Visual Analog Scale for pain, the Vancouver Scar Scale for scar evaluation, and the relapse rate at 6 months. Secondary end point included the identification of variables associated with the healing time.

**RESULTS:**

The mean time to healing was 4.7 ± 1.9 weeks in the CO_2_ laser group and 10.9 ± 4.1 weeks in the surgical deroofing group (*p* < .01). Pain score at the first dressing change was lower in the CO_2_ laser group, with a mean Visual Analog Scale score of 1.7 ± 0.8 in the CO_2_ laser group and 4.9 ± 1.7 in the surgical deroofing group (*p* < .01). The mean scar evaluation score using the Vancouver Scar Scale at 6-month follow-up was 2.5 ± 1.3 in the CO_2_ laser group and 3.4 ± 1.1 in the surgical deroofing group. The number of postprocedural complications was low in both groups (1 in the CO_2_ laser group and 3 in the surgical deroofing group). The proportion of patients achieving complete healing of the tunnels at 6 months was similar among the CO_2_ laser and the surgical deroofing group (90% in the CO_2_ laser group vs 80% in the surgical group).

**CONCLUSION:**

CO_2_ laser is a safe and effective treatment for HS tunnels, with fast healing rates and low pain perception.

Hidradenitis suppurativa (HS) is a chronic inflammatory skin disease that affects the follicular units mainly located in the apocrine glands–bearing area, including the armpits, inguinal, and perianal regions.^1^ The disease is characterized by the formation of painful nodules, abscesses, and tunnels. Hidradenitis suppurativa has a significant impact on quality of life and can lead to scarring, disfigurement, and social isolation.^1^ Although the exact pathogenesis of HS is not fully understood, it is thought to involve the infundibular portion of the hair follicle, causing follicular occlusion, bacterial colonization, and immune dysregulation.^2^

Tunnels are chronic lesions with a low tendency toward spontaneous resolution. For this reason, tunnels are typically managed with surgical deroofing.^3^ It represents a minimally invasive, tissue-sparing technique, which is frequently performed due to its simplicity and favorable aesthetic outcome.^4^ In the standard deroofing procedure, tunnels' roofs are excised, and the contained sanguinolent and gelatinous material is meticulously curetted.^5^ Preservation of the tunnel floor and adjacent tissue is prioritized, thus promoting rapid wound reepithelialization through the activation of hair follicle stem cells and sweat glands.^6^ After the deroofing procedure, wounds are intentionally left open to undergo healing by secondary intention.^4^

Van der Zee and colleagues investigated 44 consecutive patients, collectively undergoing 88 lesion deroofing procedures. Seventeen percent of the treated lesions showed recurrence within a median duration of 4.6 months, while 73% of lesions did not exhibit recurrence at a median follow-up of 34 months. The study reported a high degree of satisfaction with a rate of 90%.^7^

However, the procedure can be painful and may require general anesthesia or sedation. Also, there is a risk of wound healing complications such as infection, dehiscence, and delayed healing.^8^

CO_2_ laser treatment has emerged as a potential alternative to surgical deroofing for HS tunnels.^9^ The CO_2_ laser acts as a hydro-scalpel, vaporizing the tunnel and underlying tissue, thus creating a clean wound bed that can heal by secondary intention. The CO_2_ laser technique has been found to provide rapid procedural speed, effective management of bleeding, and reduced pain intensity compared to traditional surgery.^10^ This technique also provides the advantage of conducting most procedures under local anesthesia, thereby mitigating the risks associated with general anesthesia.^11^ Furthermore, CO_2_ laser has a bactericidal effect, which may reduce the risk of infection.^12^

There is limited research on the efficacy of CO_2_ laser treatment for HS tunnels, and no studies have directly compared the laser to surgical deroofing.

In this retrospective study, the authors aimed to compare the efficacy of CO_2_ laser treatment versus surgical deroofing for the treatment of HS tunnels. The authors aimed to explore whether CO_2_ laser treatment could demonstrate comparable efficacy to surgical deroofing in terms of healing time, incidence of complications, and cosmetic results.

## Materials and Methods

A retrospective chart review was conducted on all patients diagnosed with HS tunnels who underwent CO_2_ laser treatment between January 2022 and December 2022. The study protocol conformed to the ethical guidelines of the 1975 Declaration of Helsinki. These data were paired with records of consecutive patients who underwent surgical deroofing during the same period across 3 university centers (University Dermatology Departments of Bologna, Pisa, and Trieste). Eligible patients included those with HS tunnels located in the axilla and inguinocrural regions. This retrospective study included 10 patients treated with CO_2_ laser and 10 patients treated with surgical deroofing. Patients' demographics, including age, sex, and body mass index (BMI), were recorded. All patients were Hurley stage II. Only the axillary and inguinocrural regions were considered for inclusion in the study.

The severity of HS was assessed at baseline using the International Hidradenitis Suppurativa Severity Index 4. Pain and scarring were assessed using a Visual Analog Scale (VAS) and the Vancouver Scar Scale (VSS), respectively. Postoperative complications and disease recurrence were recorded.

### Treatment Protocol

**Figure 1.**
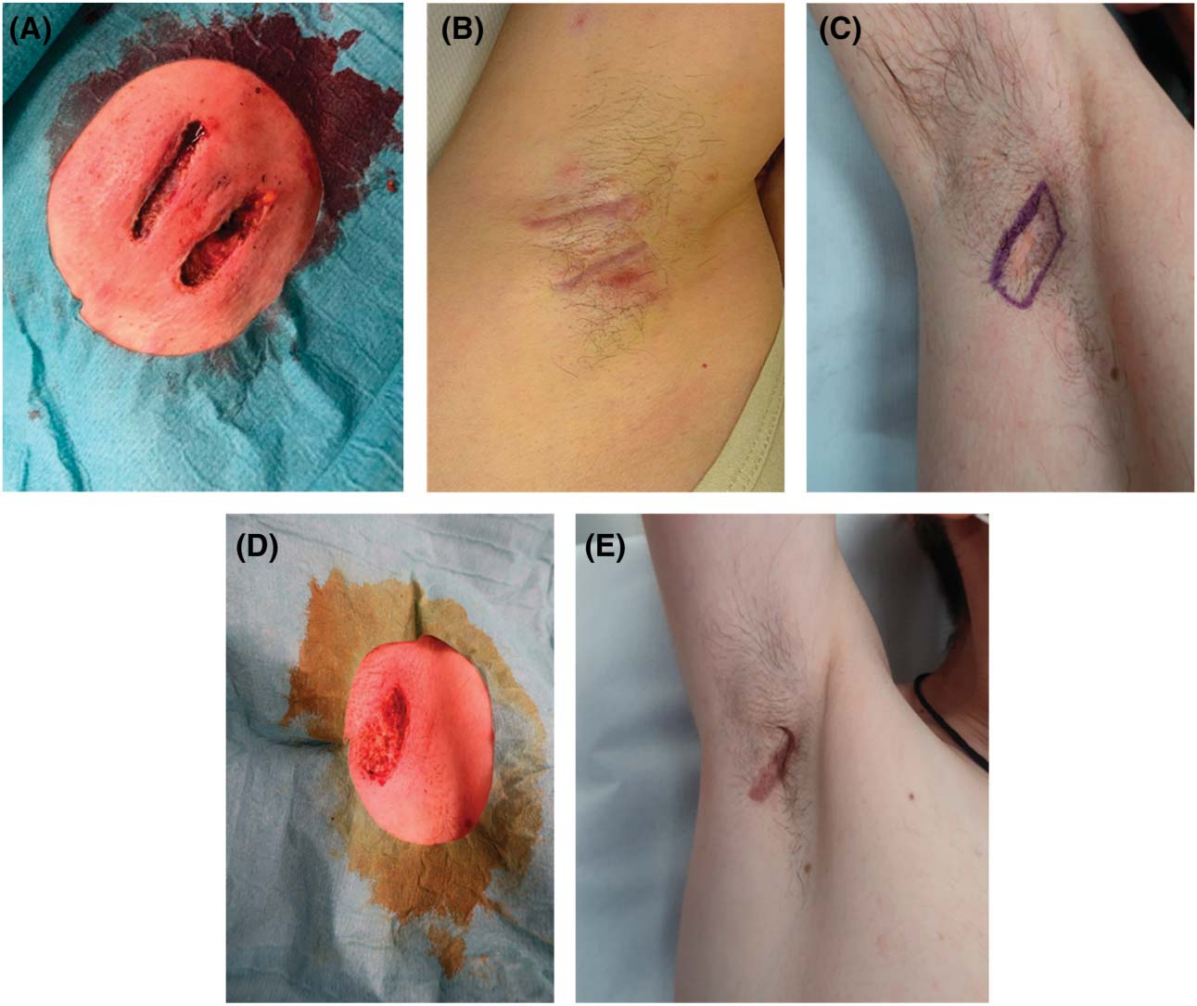
Pre- and postlaser treatment of axillary tunnels. (A and B) Immediately after and 6 months after laser treatment. (C–E) Before, immediately after, and 6 months after treatment. The Vancouver Scar Scale score for the scar at 6 months after treatment is 4: with a vascularity parameter of 1 (pink scar), pigmentation parameter of 2 (hyperpigmentation), pliability parameter of 1 (supple), and a height parameter of 0 (completely flat scar).

The CO_2_ laser treatment protocol involved the use of a 10,600 nm laser with a continuous wave mode at 4 to 10 W to perform a thorough debridement of the affected tissue (Figure 1). Blood vessels were coagulated using the defocused mode. Silver-based absorbable dressing was applied and changed every 2 days until complete healing was achieved.

**Figure 2.**
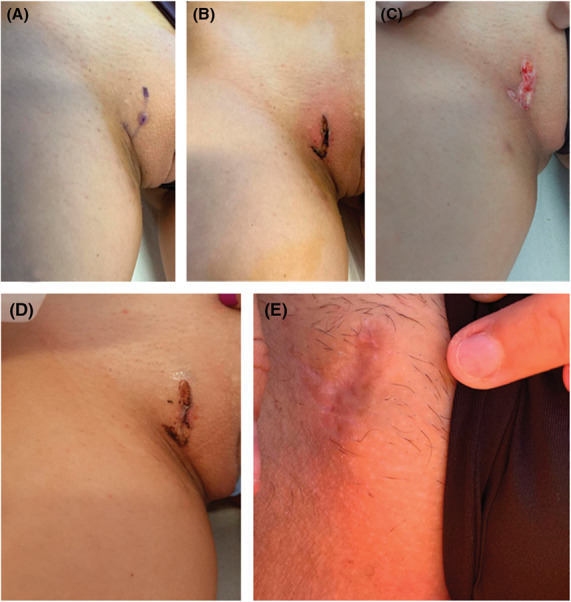
Surgical treatment of vulvar hidradenitis suppurativa tunnel. (A) Before treatment, (B) immediately after treatment, (C) 48 hours after treatment, (D) 2 weeks after treatment, and (E) 6 months after treatment.

The surgical deroofing protocol involved the use of a scalpel to open the tunnel roof followed by curettage of the tunnel while sparing its floor (Figure 2). Diathermy was used to control bleeding. The wound was left to heal by secondary intention, and silver-based absorbable dressing was applied until complete resolution.^13,14^

### Outcome Measures

Primary outcomes included the proportion of patients who achieved complete healing of HS tunnels at 6-month follow-up, VAS scores for pain assessment at the first dressing change, VSS scores for scar evaluation, and the healing time. Complete healing was defined as the clearance of draining tunnels and their substitution with scar tissue without the need for further surgical intervention, along with the absence of disease recurrence in the treated area at 6-month postoperation.

Variables associated with the healing time were investigated as a secondary end point.

### Statistical Analysis

Frequencies were used to summarize qualitative data, while means and standard deviations were used for quantitative data. For the comparison of quantitative variables, Student *t*-test or Mann–Whitney test for independent means was applied based on the parametric or nonparametric distribution, determined through Kolmogorov–Smirnov tests. The Pearson correlation coefficient was obtained to assess the linear relationship between variables. Subsequently, odds ratios (OR) along with their 95% confidence intervals, derived from univariate and multivariate linear logistic regression with healing time set as the dependent variable, were computed. Statistical analyses were performed using IBM SPSS Statistics for Windows, version 23 (IBM Corp, Armonk, NY).

## Results

### Patient Characteristics

A total of 20 patients were included in the study, with 10 patients in the CO_2_ laser group and 10 patients in the surgical deroofing group. In the CO_2_ laser group, the mean age was 30.6 years (range 21–41 years), while in the surgical group, it was 24.9 years (range 18–37 years). There were 6 females and 4 males in the deroofing group, while the CO_2_ laser group consisted of 3 females and 7 males. The baseline International Hidradenitis Suppurativa Severity Index 4 scores in the CO_2_ laser and surgical groups were 4.6 ± 3.6 and 4.3 ± 3.6, respectively. Tunnels eligible for inclusion exhibited either an absence or mild presence of inflammatory signs at the time of the procedure. Regarding medical therapies, in the CO_2_ laser group, 4 patients received adalimumab (40 mg/wk), 5 received topical high-potency corticosteroids combined with clindamycin (300 mg twice daily), and 1 received intralesional steroid injection. In the surgical deroofing group, 1 patient received clindamycin (300 mg twice daily), 2 were treated with adalimumab (40 mg/wk), 2 received topical high-potency corticosteroids combined with clindamycin (300 mg twice daily), and 3 were treated with intralesional steroid injection. Table 1 summarizes the patient characteristics of the 2 groups.

**TABLE 1.** Clinical and Demographic Characteristics of the Two Treatment Groups

	Patient No.	Smoking Status (Current or Former)	Age (yr)	Sex	IHS4 at Treatment	Ongoing Medical Therapies	BMI
Surgical deroofing	1	No	20	F	7	CLN	27.6
	2	Yes	32	F	11	ADA	22.3
	3	No	18	M	3	ADA	23.4
	4	No	23	F	5	THC + CLN	20.4
	5	No	22	F	1	THC + CLN	22.6
	6	No	30	F	5	ILK	25.4
	7	No	30	M	1	ILK	24.8
	8	Yes	37	F	0	None	29.8
	9	No	19	M	2	ILK	21.1
	10	Yes	18	M	8	None	26.2
		7 nonsmokers, 3 smokers	Mean: 24.9	Ratio (M:F): 4:6	Mean: 4.3	—	Mean: 24.36
Laser	1	Yes	27	F	13	ADA	29.3
	2	Yes	28	M	5	ADA	26.5
	3	No	26	M	5	THC + CLN	26.1
	4	Yes	23	M	1	THC + CLN	21.0
	5	No	21	M	6	ADA + ILK	24.5
	6	No	41	M	4	ADA	24.1
	7	Yes	35	F	6	THC + CLN	23.2
	8	Yes	37	F	2	THC + CLN	21.8
	9	No	31	M	0	ILK	19.3
	10	Yes	37	M	4	THC + CLN	22.2
		4 nonsmokers, 6 smokers	Mean: 30.6	Ratio (M:F): 7:3	Mean: 4.6	—	Mean: 23.8

ADA, adalimumab; BMI, body mass index; CLN, clindamycin; IHS4, Hidradenitis Suppurativa Severity Index 4; ILK, intralesional kenacort; THC + CLN, topical high-potency corticosteroids + clindamycin.

### Primary Outcomes

The proportion of patients who achieved complete healing of their HS tunnels at the 6-month follow-up was 90% in the CO_2_ laser group and 80% in the surgical deroofing group. In the surgical group, 1 patient experienced disease recurrence after 2 months, while another patient experienced recurrence after 3 months. In the laser group, 1 patient experienced recurrence after 1 month. Treatment outcomes in the 2 groups are summarized in table 2.

**TABLE 2.** Primary and Secondary Outcomes in the Two Treatment Groups

	Patient No.	VSS at 6 mo	Complete Healing of Sinus Tracts at 6 mo	Time to Healing (wk)	VAS Pain Score at First Dressing Change	Postoperative Complications
Surgical deroofing	1	4	Yes	6	6	No
	2	3	Yes	16	7	No
	3	4	Yes	12	6	No
	4	1	Yes	7	4	No
	5	4	Yes	4	1	No
	6	3	Yes	13	6	No
	7	3	Yes	15	4	No
	8	5	No	9	6	Yes (bleeding)
	9	4	No	13	4	Yes (superinfection)
	10	3	Yes	14	5	Yes (bleeding)
		Mean: 3.4	80%	Mean: 10.9	Mean: 4.9	3 (2 bleeding, 1 superinfection)
Laser	1	3	Yes	7	2	No
	2	2	Yes	7	1	No
	3	3	Yes	3	2	No
	4	1	No	3	2	No
	5	2	Yes	5	3	No
	6	3	Yes	6	1	No
	7	5	Yes	3	3	Yes (bleeding)
	8	4	Yes	7	1	No
	9	1	Yes	3	1	No
	10	1	Yes	3	1	No
		Mean: 2.5	90%	Mean: 4.7	Mean: 1.7	1 (bleeding)

VAS, Visual Analogue Scale; VSS, Vancouver Scar Scale.

The mean time to healing was statistically significant between the groups, with 4.7 ± 1.9 weeks in the CO_2_ laser group and 10.9 ± 4.1 weeks in the surgical deroofing group (*p* < .01).

The mean VAS pain score recorded at the time of the first dressing change was 1.7 ± 0.8 in the CO_2_ laser group and 4.9 ± 1.7 in the surgical deroofing group (*p* < .01).

The mean scar evaluation score using the VSS at the 6-month follow-up was 2.5 ± 1.3 in the CO_2_ laser group and 3.4 ± 1 in the surgical deroofing group (*p* = .11).

Subsequently, univariate and multivariate linear regressions were conducted to analyze the aforementioned predictors of healing time. The VAS score at the first medication was the sole significant predictor, showing a significant correlation in both univariate analysis with an OR of 0.7 (CI: 0.2–2.5) and multivariate analysis with an OR of 0.6 (CI: 0.4–1.8).

### Secondary Outcomes

Postoperative complications were recorded in both treatment groups. In the CO_2_ laser group, 1 patient (10%) experienced postoperative complications (bleeding). There were no cases of wound infection in the CO_2_ laser group. In the surgical deroofing group, 3 patients (30%) experienced postoperative complications, including 1 case of wound infection and 2 cases of bleeding.

No significant correlation was identified between smoking status or BMI and the incidence of postoperative complications.

A significant positive correlation was found between pain intensity, measured by the VAS at the first dressing change, and healing time in weeks, as determined by a Pearson correlation coefficient of 0.62 (*p* = .03). This association remained significant in the authors' multivariate analysis.

In addition, a significant correlation was observed between VSS and smoking, with a Pearson coefficient of 0.55 (*p* = .01). Furthermore, there was a correlation between VSS and VAS at the first dressing change, with a Pearson coefficient of 0.56 (*p* = .008).

## Discussion

The primary aim of this study was to compare the outcomes of CO_2_ laser treatment versus surgical deroofing for HS tunnels. Remarkably, a high percentage of patients achieved complete healing in both the laser and surgical groups, with 80% achieving full recovery in the surgical group and 90% in the laser group. Although no statistically significant difference in the proportion of patients achieving complete healing was observed between the 2 groups, the mean time to healing was significantly shorter in the CO_2_ laser group compared to the surgical deroofing group (*p* < .01).

To date, no studies have directly compared the efficacy, in terms of achieving complete healing and evaluating healing times, between these 2 techniques. Surgical deroofing is considered one of the most effective methods to treat HS tunnels.^7^ This method involves the exposure and removal of epithelialized tunnels and associated keratinous debris, which not only halts scarring but also helps prevent the disease recurrence.^10^ More recently, CO_2_ laser treatment has demonstrated efficacy in managing HS tunnels. This technique is characterized by lower invasiveness and enhanced control over bleeding.^15,16^ In addition, high patient satisfaction has been reported.^17^ In the authors' study, although there was no statistically significant difference in terms of complete healing at 6 months, suggesting comparable effectiveness between the 2 treatments, there were faster healing times for the CO_2_ laser. One limitation of the laser technique is a relatively high recurrence rate, reported as high as 29%,^17^ while a recurrence rate of 17% has been reported for surgical deroofing.^7^ In the authors' series, only 1 patient experienced disease recurrence in the laser group, whereas 2 patients relapsed in the surgical group. A limitation of the authors' study is that recurrences were detected within a maximum of 6-month postprocedure, and longer follow-up may be necessary to determine the actual recurrence rate. In addition, not all tunnels underwent ultrasonographic evaluation, which could influence the recurrence rate due to differences in size, depth, and fibrosis degree of the tunnels.

In advanced cases of HS, Hurley Stage III or greater, wide local excision (WLE) stands as a viable surgical option, albeit more invasive and complex compared to deroofing.^4^ This technique involves the complete removal of all affected tissue, including the skin, subcutaneous fat, nodules, and tunnels. Excision extends down to the deep fascia, ensuring a clearance margin of 1 to 2 cm. Subsequent wound management options include primary closure, secondary intention healing, or closure using a flap or skin graft.^18^ In a comprehensive meta-analysis conducted by Mehdizadeh and colleagues,^19^ a 13% recurrence rate was observed among patients undergoing WLE, while those who had local excision experienced a 22% recurrence rate, and those who underwent deroofing had a recurrence rate of 27%. In addition, the study highlighted a comparatively higher recurrence rate of 15% after WLE with primary closure, in contrast to 8% with flaps and 6% with grafts.

Although WLE appears to have a lower recurrence rate, it is associated with a longer healing time, a more complex surgical technique, and a more demanding postoperative management compared to both surgical deroofing and laser treatment.

A novel surgical technique for managing chronic lesions of HS is Skin-Tissue-sparing Excision with Electrosurgical Peeling. Skin-Tissue-sparing Excision with Electrosurgical Peeling is considered a tissue-preserving surgical approach for Hurley stage II/III HS, where fat is maximally spared through successive tangential excisions of lesional tissue until reaching the epithelialized bottom of tunnels. In addition, this method thoroughly removes fibrotic tissue, thereby reducing the risk of recurrence.^20^ Direct comparisons of the efficacy and recurrence rates of this new technique with WLE or other surgical or laser approaches are currently lacking.

In terms of pain, the CO_2_ laser group had significantly lower VAS pain scores at the time of the first dressing change compared to the surgical deroofing group at the time of the first dressing change (*p* < .01). This is likely due to the fact that CO_2_ laser treatment is associated with less tissue trauma and postoperative pain compared to surgical deroofing. Although direct comparisons between the pain experienced during surgical deroofing and CO_2_ laser procedures in HS tunnels are lacking, numerous studies in other surgical contexts have consistently reported lower levels of pain and discomfort associated with CO_2_ laser surgery when compared to scalpel surgery.^21–23^

The scar evaluation scores were not significantly different between the 2 groups at 6-month follow-up, although there was a tendency toward better aesthetic results in the CO_2_ laser group (mean VSS of 2.5 ± 1.3 in the CO_2_ laser group and 3.4 ± 1.1 in the surgical deroofing group, *p* = .11). There are no studies in the literature that assess the aesthetic outcome of scars from chronic HS lesions using validated scar scales, nor are there direct comparisons between CO_2_ laser and scalpel surgery in this regard. Nevertheless, a prior study conducted by Finley and Ratz^15^ involving 7 patients with HS, who underwent CO_2_ laser excision with healing by second intention, reported positive cosmetic outcomes in all cases, as assessed by both patients and operators.

A recent study investigating the use of CO_2_ laser excision in late-stage HS examined the incidence of keloid formation.^24^ The study found that none of the treated patients, including those of African descent with a history of keloids/hypertrophic scars (patients at higher risk), developed this complication. This observation may suggest that CO_2_ excision does not increase the risk of keloid development. Similarly, in the authors' cohort, none of the patients treated with CO_2_ laser developed keloids or hypertrophic scars.

It has been hypothesized that known comorbidities and habits associated with HS, specifically smoking and obesity, may influence postprocedural complications, leading to an increased rate of complications and prolonged healing times. However, the authors' study did not reveal any correlation between the occurrence of postprocedural complications and smoking status or BMI. This is consistent with the findings of the systematic review by Bouazzi and colleagues,^25^ where no high-quality data were identified supporting an association between complication rates after surgical interventions among comparable patients with HS, irrespective of smoking and obesity status.

## Conclusion

In conclusion, the results of this study suggest that CO_2_ laser and surgical deroofing are both effective treatments for HS tunnels, with comparable rates of complete healing and cosmetic outcomes. However, CO_2_ laser treatment is associated with faster healing times and less postoperative pain compared to surgical deroofing. These findings may have important implications for the management of HS tunnels, and further research is needed to confirm these results.

### Limitations of the Study

Limitations of the study include the small sample size for both treatment groups, necessitating further trials with larger cohorts to assess the efficacy and adverse event rates of the 2 treatments more comprehensively. In addition, not all tunnels underwent ultrasound evaluation, despite the known advantages of ultrasound in providing a more precise characterization of chronic HS lesions compared to clinical assessment. Furthermore, the lack of data regarding the recurrence rate of the 2 techniques beyond the 6-month postprocedure period is a notable limitation, particularly given its significance in the context of HS surgery.

It is noteworthy that a higher percentage of patients in the CO_2_ laser group were undergoing biologic therapy (40%) compared to the deroofing group (20%). While biologic therapy is not associated with increased perioperative complications, it cannot be ruled out that this difference in percentages may have influenced the outcomes in the laser group.
